# Late gadolinium enhanced cardiovascular magnetic resonance of lamin A/C gene mutation related dilated cardiomyopathy

**DOI:** 10.1186/1532-429X-13-30

**Published:** 2011-06-20

**Authors:** Miia Holmström, Sari Kivistö, Tiina Heliö, Raija Jurkko, Maija Kaartinen, Margareta Antila, Eeva Reissell, Johanna Kuusisto, Satu Kärkkäinen, Keijo Peuhkurinen, Juha Koikkalainen, Jyrki Lötjönen, Kirsi Lauerma

**Affiliations:** 1 Department of Radiology, University of Helsinki and HUS Radiology (Medical Imaging Center) P.O. Box 340, FI-00029 HUS, Finland; 2 Department of Cardiology, Helsinki University Central Hospital, P.O. Box 340, FI-00029 HUS, Finland; 3 Boehringer Ingelheim Finland Ky Tammasaarenkatu 5, FI-00180 Helsinki, Finland; 4 Heart Center, Kuopio University Hospital, P.O. Box 1777, FI-70211 Kuopio, Finland; 5 VTT Technical Research Centre of Finland, P.O. Box 1300, FI-33101 Tampere, Finland

## Abstract

**Background:**

The purpose of this study was to identify early features of lamin A/C gene mutation related dilated cardiomyopathy (DCM) with cardiovascular magnetic resonance (CMR). We characterise myocardial and functional findings in carriers of lamin A/C mutation to facilitate the recognition of these patients using this method. We also investigated the connection between myocardial fibrosis and conduction abnormalities.

**Methods:**

Seventeen lamin A/C mutation carriers underwent CMR. Late gadolinium enhancement (LGE) and cine images were performed to evaluate myocardial fibrosis, regional wall motion, longitudinal myocardial function, global function and volumetry of both ventricles. The location, pattern and extent of enhancement in the left ventricle (LV) myocardium were visually estimated.

**Results:**

Patients had LV myocardial fibrosis in 88% of cases. Segmental wall motion abnormalities correlated strongly with the degree of enhancement. Myocardial enhancement was associated with conduction abnormalities. Sixty-nine percent of our asymptomatic or mildly symptomatic patients showed mild ventricular dilatation, systolic failure or both in global ventricular analysis. Decreased longitudinal systolic LV function was observed in 53% of patients.

**Conclusions:**

Cardiac conduction abnormalities, mildly dilated LV and depressed systolic dysfunction are common in DCM caused by a lamin A/C gene mutation. However, other cardiac diseases may produce similar symptoms. CMR is an accurate tool to determine the typical cardiac involvement in lamin A/C cardiomyopathy and may help to initiate early treatment in this malignant familiar form of DCM.

## Background

Dilated cardiomyopathy (DCM) is a major cause of heart failure and sudden cardiac death. About one third of DCM cases are familial. During the past few decades, several DCM disease genes have been identified, many of them limited to individuals or families [1-3]. The lamin A/C gene (LMNA) is so far the most significant disease gene for DCM. It has been estimated that LMNA mutations cause up to 10% of familial DCM. The penetrance of the LMNA mutations causing cardiomyopathy is nearly complete [4]. The gene resides on chromosome 1q21.2-q21.3. By alternative splicing, it codes for lamins A and C, proteins found in the nuclear lamina. LMNA mutations may also cause a variety of other clinical entities such as neuromuscular syndromes, lipodystrophy or premature aging disease, i.e. progeria. We have previously described several LMNA mutations among Finnish DCM patients [2,3].

Carriers of LMNA mutations causing cardiomyopathy should be recognised at an early stage so that regular cardiologic follow-up can be arranged; affected individuals have a high risk of sudden cardiac death or severe heart failure by middle age [5]. However, most of the patients are asymptomatic or only have mild symptoms for many years, and diagnosis is therefore difficult. Moreover, the serious nature of LMNA-related cardiomyopathy is easily overlooked at the early stage because the left ventricular (LV) dilatation is typically mild at the beginning, and it may remain so even later on although the LV ejection fraction may be very low. In other words, LMNA mutations may cause DCM, or cardiomyopathy with decreased systolic function, combined with LV dilatation that is too mild to fulfil the criteria of DCM. The earliest cardiac finding in cardiomyopathy caused by a LMNA mutation is usually conduction system disease, associated with atrial or ventricular arrhythmias, atrioventricular (AV) conduction abnormalities and myocardial dysfunction [6].

Echocardiography is regarded as the standard method to clinically study DCM; however, cardiovascular magnetic resonance (CMR) plays an important role in the characterisation and risk stratification of patients with DCM. Late gadolinium enhancement (LGE) CMR with standard cine-imaging is an accurate and reproducible method both to demonstrate myocardial fibrosis and to measure wall thickness as well as global and regional function of both ventricles [7,8].

The main purpose of this study was to characterise myocardial fibrosis, regional wall motion abnormalities, ventricular dilatation, longitudinal LV systolic function and global function with LGE CMR in asymptomatic or mildly symptomatic carriers of LMNA mutations causing DCM. In addition, we investigated the possible association between the localisation of myocardial fibrosis and the conduction abnormalities documented with electrocardiography (ECG).

## Methods

### Study patients

#### LMNA mutation carriers

The LMNA mutations had originally been identified in DCM patients who participated in an earlier molecular genetic study of DCM carried out in collaboration between the Helsinki University Hospital and the University of Kuopio [2,3]. The subjects in the current study, recruited from the families of these DCM patients, were carriers of an LMNA mutation. The study group comprised seventeen subjects (eight men and nine women, mean age 36 ± 12, 39 ± 18 years, body surface area (BSA) 1.8 ± 0.2 m^2^) recruited for this study between 2004 and 2008. Each subject was a heterozygote for one mutation. A summary of the mutations, symptoms and ECG findings are presented in Table 1. The clinical evaluation of the subjects comprised hospital records, clinical examination, venous blood samples, 12-lead ECG, echocardiography and CMR. CMR was performed during the first visit.

**Table 1:** The clinical characteristics of LMNA mutation carriers.

LMNA carrier	Gender (male/female)	Age (years)	Mutation	Symptoms	Cardiac medication	Rhythm	PR (upper limit 200 ms)	QRS (upper limit100 ms)	**q-waves**, low r-waves
1	M	42	ser143pro	vertigo	ACE-blocker	fa	-	158	V1-3 QS

2	F	54	ser143pro	dyspnea extrasystolia	none	sr	276	124	no

3	F	30	ser143pro	none	none	sr	236	96	no

4	M	32	ser143pro	none	none	sr	176	112	no

5	M	18	ser143pro	palpitation	none	sr	176	96	no

6	F	18	ser143pro	none	none	sr	164	108	no

7	F	29	ser143pro	palpitation	none	sr	182	92	no

8	M	39	T1085Xdeletion	none	none	sr	322	106	no

9	M	25	ala132pro	paroxysmal flutter	warfarin, bisoprolol	sr	176	112	no

10	F	52	ala132pro	extrasystolia	none	sr	276	92	no

11	M	29	arg190trp	palpitation	none	sr	160	100	no

12	F	32	arg190trp	palpitation	none	sr	140	84	no

13	F	64	arg190trp	vertigo	warfarin, bisoprolol, digoxin	fa	-	84	Low r-waves V1-4

14	M	58	arg190trp	dyspnea NYHA 2	warfarin, AT-receptor blocker, diuretic, betablocker	sr	208	100	QS V1

15	M	43	ser143pro	none	none	sr	328	90	no

16	F	35	ser143pro	extrasystolia	none	sr	224	76	no

17	F	39	ser143pro	none	none	sr	254	114	QS V1-2

This table demonstrates patients age, gender, type of lamin A/C mutation, symptoms, medications, heart rhythm and possible abnormalities in ECG (prolonged PR interval, widened QRS complex or presence on q-waves or low t-waves).

NYHA = New York Heart Association classification; sr = sinus rhythm, fa = atrial fibrillation

All participants gave written informed consent. The project was approved by the local institutional ethics committee.

### CMR

CMR was performed with a 1.5 T MR imager (Avanto; Siemens, Erlangen, Germany) using a multi-channel body-array coil as a receiver. Breath-hold cine CMR was performed using retrospectively electrocardiographically gated segmented true fast imaging with steady-state free precession (SSFP). Cine MR images were obtained in vertical and horizontal long-axis, and a stack of images in short-axis plane covering both ventricles was acquired. The imaging parameters were TR/TE 3.0/1.6 msec, flip angle 52 degrees, 256 × 256 matrix, 240 × 340 mm field of view. Slice thickness was 6 mm and interslice gap 20%. The temporal resolution was 42-49 msec.

Fi**v**e to fifteen minutes after the injection of a contrast agent (gadoterate meglumine, Dotarem® 0.1 mmol/kg) LGE images were acquired in the same views as for cine images, using inversion recovery turbo fast-low angle shot (FLASH). The imaging parameters were TR/TE 2.58/2.3 msec, flip angle 50 degrees, 256 × 256 matrix, 240 × 340 mm field of view. Slice thickness was 8 mm and interslice gap 0%. Inversion time was optimised for each measurement to null the signal intensity of normal myocardium (240-360 msec).

### Image analysis

The images were analysed in consensus by two experienced (> 10 years experience) cardiac radiologists (SK, MH). In each patient, the three LV short axis sections were divided into 17 segments according to American Heart Association (AHA) guidelines [9]. The planimetry of enhancing area was calculated within each segment. The segmental extent of enhancement on LGE images was graded using the following score: 0 = no enhancement, 1 = 1-25% enhancement, 2 = 26-50% enhancement, 3 = 51-75% enhancement, 4 = 76-100% enhancement. Right ventricle (RV) enhancement was also determined from the LGE images.

On the cine images, segmental wall motion was visually assessed and scored as 1 = normal, 2 = hypokinesia, 3 = akinesia and 4 = dyskinesia.

Measurement of volumes and ejection fraction of both ventricles were segmented semi-automatically by tracking the endocardial borders of both ventricles with a segmentation tool developed for this purpose [10].

To assess longitudinal LV systolic function, mitral annular displacement (MAD) was measured as the difference (between end-diastole and end-systole) of the distance from apex to the lateral and septal sites of the mitral annulus in the horizontal long-axis view, and apex to the anterior and inferior sites in the vertical long-axis view. The mean value of MAD at all four sites was then calculated.

### Statistical analysis

Statistical analysis was performed using NCSS version 2007 software. Chi-Square test and Fisher's Exact test were used to calculate for correlation between the percentage of LGE and wall motion abnormalities. Volumetric results of both ventricles were compared to normal values published earlier by Maceira et al [11,12] whose cine sequence technique (SSFP), protocol as well as volumetric analysis were comparable to those used in our study. Relationships between MAD and EF as well as MAD and percentage of LGE were assessed using Spearman Correlations test. Correlation between conduction defects and myocardial fibrosis was calculated using t-test.

## Results

**Figure 1:**
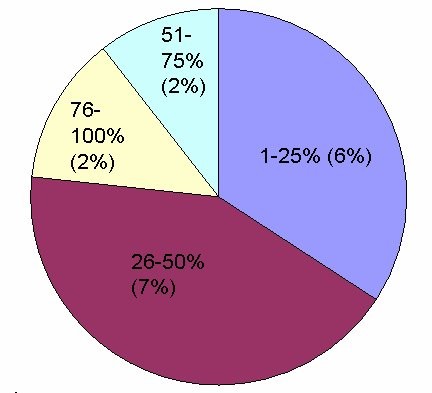
**The extent of enhancement (%) in abnormal myocardial segments**. The percentage of late gadolinium enhancement in involved myocardial segments (n = 47). The extent of enhancement was under 25% in sixteen (6%) segments. Twenty (2%) of the segments showed enhancement of 26-50%. Eleven segments (4%) enhanced over 50% of the area.

**Figure 2:**
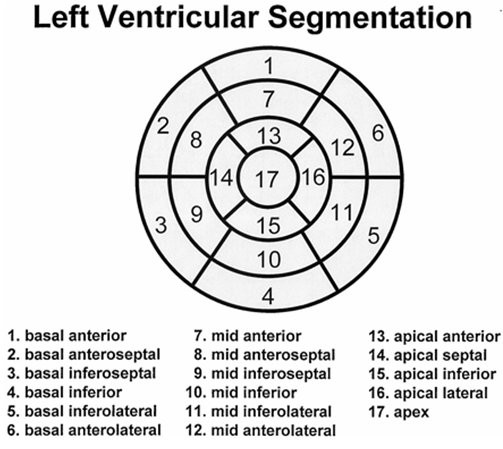
**Left ventricular segmentation**. The left ventricle was divided into 17 segments according to American Heart Association.

**Figure 3:**
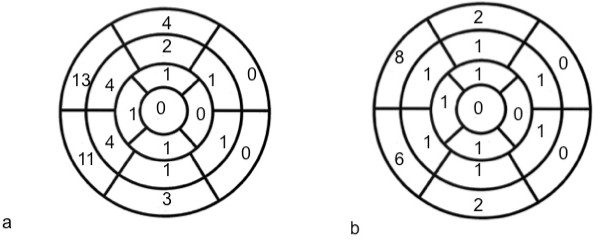
**Correlation between enhanced myocardial segments and wall motion abnormalities**. Distribution of enhanced segments (a) and wall motion abnormalities (b) of the left ventricle are shown in bull's eye plots. Our data demonstrates predominant involvement of fibrosis and wall motion disturbances in the basal and anteroseptal segments.

**Figure 4:**
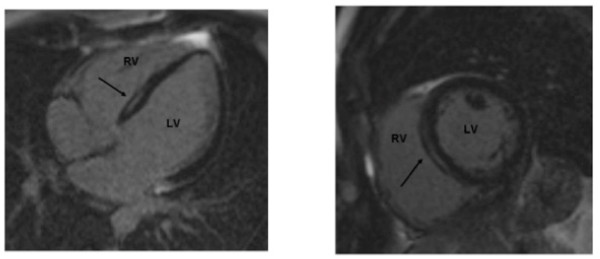
**Myocardial late gadolinium enhancement (LGE) in the septal region evaluated with cardiovascular magnetic resonance (CMR)**. LGE CMR of a 32-year old male with lamin A/C mutation DCM. Four-chamber (a) and short axis (b) views of the heart show typical mid-myocardial and linear enhancement of the basal septum (arrows).

Out of 17 patients, 15 exhibited LGE. Among the total of 289 segments, LGE was observed in 47 (16%). The extent of LGE was less than 50% in 36/47 enhanced segments (Figure 1). In all patients, LGE occurred in the basal or mid-ventricular septal wall (Figure 2, 3). RV free wall enhancement was not observed. Thirty three of the 47 enhanced segments were in the septal area (segments 2-3, 8-9, 14). The typical pattern of LGE was linear and mid-myocardial (Figure 4). Diffuse enhancement of LV myocardium was observed in two advanced cases of the disease. LV wall thickness was normal in all patients. Abnormal motion was found in 27 segments (9%). The motion abnormalities were predominantly located in the basal regions of LV (segments 1-4). There was hypokinesia in 17 segments and akinesia in 10 segments. The correlation between the extent of enhancement and abnormal motion was significant (p < 0.001). Abnormal motion was found in 50% of segments enhancing 26-50% and in all segments if the enhancement was over 50% (Table 2).

**Table 2:** Myocardial late gadolinium enhancement (LGE) and movement abnormalities in the left ventricle.

Percentage of LGE within the segment	Normal movement (%)	Abnormal movement (%)	Total
No enhancement	240 (99)	2 (1)	242

0-25%	12 (75)	4 (25)	16

26-50%	10 (50)	10 (50)	20

51-75%	0 (0)	6 (100)	6

76-100%	0 (0)	5 (100)	5

total	262	27	289

LGE correlated significantly (p < 0.001) with abnormal movement analyzed in each 17 myocardial segment separately. Abnormal motion (hypokinesia or akinesia) was documented in 27 segments out of 289 segments. Abnormal motion was found in 50% of segments enhancing 26-50% and in all segments if enhancement was over 50%.

All patients with conduction abnormalities (11 with an AV conduction defect and two with a widened QRS complex) and two with atrial fibrillation showed enhancement of the basal and mid-ventricular septum. Conduction abnormalities correlated significantly with presence of LGE in the LV septum (p = 0.01). Four patients had a normal ECG recording, and two of them had enhancement of the basal septum. Table 3 summarises the ECG abnormalities in relation to the enhanced segments and regional wall motion abnormalities.

**Table 3:** Electrocardiography (ECG) and cardiovascular magnetic resonance (CMR) findings.

Patient gender	Age	ECG	Enhanced myocardial segments	Abnormal movement (segments)	LV EDV ml/m^2^	LV ESV ml/m^2^	LV EF %	RV EDV ml/m^2^	RV ESV ml/m^2^	RV EF %	MAD cm
M	42	fa	1-3	2-3	**112**	**84**	**25**	65	**49**	**26**	**0.73**

F	54	DAV 1	2-3	2-3	**107**	**50**	**53**	**97**	**40**	59	**0.93**

F	30	DAV 1	2,8		**106**	**37**	65	97	38	61	1.61

M	32	sr qrs > 100 ms	2-4	2-3	**109**	**44**	60	93	38	59	1.51

M	18	sr			95	**45**	**53**	95	48	49	1.85

F	18	sr qrs > 100 ms	2		83	30	64	76	33	55	1.60

F	29	sr	3		80	29	64	75	28	63	**1.28**

M	39	sinusbrady DAV 1	2-3		**112**	**41**	63	**120**	**54**	56	2.13

M	25	sinusbrady qrs > 100 ms	2-3,7-9	2	**117**	41	65	**123**	49	60	1.68

F	52	DAV 1	1-2		76	**34**	**56**	63	32	**50**	1.55

M	29	sr			98	37	62	95	42	56	1.78

F	32	sr	2-3,9								**1.32**

F	64	fa	2-4,8-9,11-12	2-4	74	**36**	**52**	67	37	**45**	**0.97**

M	58	DAV 1	1-4,7-10,13-15	1-4,7-15	**118**	**59**	**50**	100	**58**	**41**	**0.72**

M	43	DAV 1	2-3	2-3	**117**	**68**	**42**	71	**48**	**33**	**1.08**

F	35	DAV1	1-2	1-2	73	26	65	61	28	54	**1.43**

F	39	DAV 1	1-17		88	32	64	75	26	66	**1.44**

This table demonstrates ECG and CMR findings of lamin A/C dilated cardiomyopathy patients. Type of conduction defect documented with ECG, volumetric and functional data of ventricles, late gadolinium enhancement and wall motion abnormalities of myocardial segments evaluated with CMR are presented. Abnormal volumetric and functional values are shown highlighted.

RV = right ventricle, LV = left ventricle, EDV = end diastolic volume, ESV end systolic volume, EF = ejection fraction fa = atrial fibrillation, sr = sinus rhythm, DAV = atrio-ventricular block, MAD = mitral annular displacement (normal value between 1.5-1.6 cm)

A volumetric analysis of one female subject was excluded because of poor cine image quality due to arrhythmia. When compared with the age, sex and BSA standardised normal values published earlier by Maceira et al [8,9], ventricular volumes and systolic function were preserved in five patients. Minor systolic dysfunction without ventricular dilatation was observed in three patients (LV dysfunction in one patient and dysfunction of both ventricles in two patients). Mild LV dilatation without systolic dysfunction was observed in four patients, two of whom also had a slightly dilated RV. A dilated LV and systolic dysfunction was observed in four patients, three of whom also had RV dysfunction, and one female subject had RV dilatation without dysfunction. Table 4 summarises the global ventricular functions and ejection fractions for each of the 16 patients.

**Table 4:** Left ventricular volumes and function parameters evaluated with cardiovascular magnetic resonance.

	Males n = 8	Females n = 8
**BSA m^2^**	1.99 ± 0.20	1.80 ± 0.25

**LV EDV ml/m^2^**	110 ± 9 (64-103)	86 ± 12 (59-99)

**LV ESV ml/m^2^**	52 ± 16 (17-41)	34 ± 4 (17-37)

**LV SV ml/m^2^**	57 ± 15 (42-68)	52 ± 10 (14-37)

**LV EF %**	52 ± 13 (57-75)	60 ± 0.1 (56-77)

**RV EDV ml/m^2^**	95 ± 20 (60-106)	76 ± 14 (55-92)

**RV ESV ml/m^2^**	48 ± 6 (14-43)	33 ± 5 (12-38)

**RV SV ml/m^2^**	47 ± 20 (38-70)	43 ± 12 (36-60)

**RV EF %**	47 ± 13 (53-78)	57 ± 7 (54-78)

Left ventricular volumes and function parameters of 16 patients with dilated cardiomyopathy, who were heterozygotes with one LMNA mutation. Cine images of one patient were excluded from volumetric analysis for poor image quality. Normal values are presented within parenthesis.

BSA = body surface area, RV = right ventricle, LV = left ventricle, EDV = end diastolic volume, ESV = end systolic volume, EF = ejection fraction

Longitudinal LV systolic function was decreased in nine patients when compared to normal values published by Nikitin et al [13]. Decreased MAD was observed in eight patients with decreased EF, three patients (18%) with normal EF showed longitudinal LV systolic dysfunction (Table 3).

**Figure 5:**
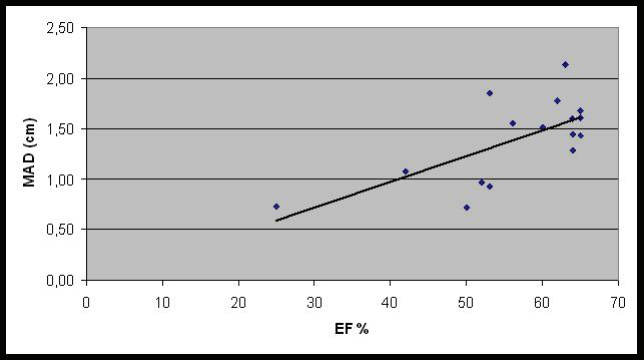
**Relationship between mitral annular displacement (MAD) and Left ventricular ejection fraction (EF)**. Longitudinal LV systolic function measured using MAD showed correlation with decreased EF. Nine patients had decreased MAD (normal value 1.5-1.6 cm). Three lamin A/C DCM patients with normal EF had decreased MAD as an early marker of systolic dysfunction.

**Figure 6:**
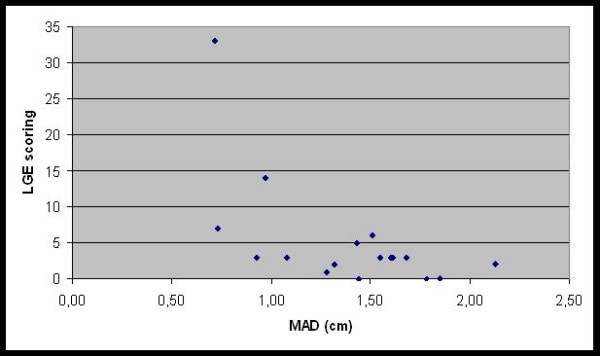
**Relationship between mitral annular displacement (MAD) and late gadolinium enhancement (LGE)**. Longitudinal LV systolic function measured using MAD showed a significant correlation with a percentage of LGE (fibrosis) in the left ventricle myocardium. LGE scoring was calculated by summarizing enhanced LV segments of each patient. LV = left ventricle.

MAD was significantly lowered when EF was decreased (p < 0.03) (Figure 5). MAD correlated significantly with percentage of myocardial LGE (p < 0.01) (Figure 6).

## Discussion

The present study demonstrates that 88% of asymptomatic or mildly symptomatic carriers of LMNA mutations causing cardiomyopathy had typical myocardial fibrosis, predominantly in the mid-myocardium of the basal septum. This was observed in all individuals with an AV conduction defect. The pattern of enhancement was typically linear and less than 50% of the area of the segment. Enhancement was associated with wall motion abnormalities at LV basal segments, where the degree of enhancement had a significant correlation with decreased motion. Minor systolic dysfunction without ventricular dilatation, preserved systolic function with mild LV dilatation or systolic dysfunction with mild dilatation of LV, or of both ventricles, was observed in 69% of LMNA mutation carriers. In our patients as an early marker of the disease longitudinal LV systolic function was decreased in nine patients when compared to normal values [13]. In addition, we found that LV EF as well as percentage of myocardial fibrosis correlated significantly with abnormal MAD.

Early studies show that even a very small amount of myocardial scar tissue is visible with routine LGE CMR sequences [14]. Myocardial diseases have typical enhancement patterns on the basis of their characteristic pathology [15]. In previous studies, about one third of DCM patients have demonstrated mid-myocardial linear enhancement on LGE CMR in a non-coronary distribution, predominantly in the LV basal and mid-ventricular septum [8,16-19]. However, Ebeling Barbier et al have documented that 30% of elderly volunteers without or with minimal coronary artery disease showed myocardial LGE, but mostly inferolateral region of the LV. On the other hand, Breuckmann et al found that 12% of marathon runners had LGE. Subendocardial and transmural LGE pattern was found in five marathon runners, but also non-CAD pattern was documented in seven subjects [20]. A healthy control group of DCM patients in study by Zimmerman et al [18] showed no myocardial LGE. As LGE imaging is widely used as a gold standard in cardiac research, we did not collect any new data of healthy subjects [21]. In our study, 88% of LMNA mutation carriers had typical myocardial fibrosis, predominantly mid-myocardially in the basal septum. High degree of LGE in our patients may reflect the effect of these mutations alone and not the effects of Lamin A/C mutations in general.

Assomull et al reported that mid-wall myocardial fibrosis in DCM patients predicted both ventricular tachycardia and sudden cardiac death [17]. Cardiomyopathy caused by a LMNA mutation has a malignant course and affected individuals are at high risk of sudden cardiac death [6]. The earliest cardiac finding is usually conduction system disease [22]. Raman et al demonstrated mid-myocardial fibrosis and abnormal LV diastolic function with LGE CMR in patients with DCM caused by a LMNA mutation [23]. This is in line with our findings, the majority of our patients having myocardial scarring and regional motion abnormalities in corresponding areas.

Previous autopsy studies have shown the location of fibrosis near the region of the conduction system and this has been postulated to be the cause of the AV blocks in LMNA-related cardiomyopathies [24,25]. In consensus, cardiac conduction defects such as a prolonged PR interval, a widened QRS complex and atrial fibrillation correlated with the presence of septal wall fibrosis and motion abnormalities in our study.

Zimmermann et al concluded that enhancement is a typical feature on CMR carried out in DCM patients but it did not correlate with myocardial biopsy findings [18]. Since a biopsy only yields information about the selected biopsy site the detection of fibrosis with biopsy can be unsatisfactory due to the mid-myocardial location of fibrosis. Whereas LGE imaging can be used to reliably detect the location and extent of fibrosis, and it can even be used to offer guidance in biopsy sampling [26]. Sixty-nine percent of our asymptomatic or mildly symptomatic patients also showed mild ventricular dilatation and systolic failure in global analysis. Twelve of our 17 patients were also participants in the study of Koikkalainen et al, which determined an amalgam of parameters calculated from cine images typical for LMNA mutation carriers [27]. The study also found a significant difference between the patient and control group in volumetric and function parameters even in the early and asymptomatic stage of the disease. Fifty-three percent of our patients had decreased longitudinal LV systolic function. LV myocardial contraction and relaxation are reported to impair first in longitudinal direction in patients with heart failure [28,29].

## Conclusions

In conclusion, in the early stage of DCM caused by a LMNA gene mutation, AV conduction defects are common with or without a minor depression in LV systolic function. Usually the suspicion of DCM caused by LMNA mutations is based on the presence of conduction system disturbances in an ECG and by clinical exclusion of other aetiologies. However, other inflammatory or infiltrative cardiomyopathies may resemble DCM caused by LMNA mutations. If the typical pattern and location of myocardial fibrosis, mild LV dilatation and systolic ventricular dysfunction can be accurately observed with LGE CMR in the early stage of the disease, the diagnosis can be confirmed by DNA analyses. Myocardial scarring is known to provide a substrate for ventricular dysfunction, conduction abnormalities and ventricular arrhythmias. The disease is known to have a malignant course, and the combination of ECG recordings and LGE CMR will help to recognise these patients. LGE CMR can also be used to differentiate between LMNA-related cardiomyopathy and cardiac sarcoidosis, both of which can cause AV conduction defects and arrhythmias. Although no specific medical treatment exits so far to prevent the development of LMNA-related cardiomyopathy, sudden cardiac death may be prevented by a timely placement of a pacemaker or an implantable cardioverter defibrillator.

LGE CMR is also a reproducible method to monitor the progression of the disease and therefore we have an ongoing follow-up study with DE-MRI to obtain further information about the progression of the familial form of DCM caused by a LMNA mutation.

### Study limitations

We had a limited number of patients, all of them carriers of a rare familial form of DCM caused by a LMNA mutation (14 out of the 17 Lamin A/C mutations in the population are due to either a ser143pro mutation (n = 10) or an arg190trp mutation (n = 4)). Wall motion abnormalities were visually graded as well as the percentage of enhancement in LV segments. As both cine volumetry and LGE imaging are widely used as gold standards in cardiac research, we did not collect any new data of healthy subjects. Global volumetric CMR results and longitudinal systolic function were compared with previously published normal values obtained from healthy volunteers.

## Competing interests

The authors declare that they have no competing interests.

## Authors' contributions

MH, SK, KL have carried out cardiovascular magnetic resonance imaging studies, performed image analysis and participated drafting the manuscript. MA have been involved in cardiovascular magnetic resonance imaging and analyzing images. TH have participated in the study design and coordination, performed clinical examinations and echocardiography's of the patients and have been involved in drafting the manuscript. RJ, MK, ER, JK, SK and KP have participated in the design of the study, performed clinical examinations and echocardiography's of the patients and the control group. They have been involved in revising the manuscript. JK and JT have participated in analyzing magnetic resonance images. All authors have read and approved the final manuscript.
